# The trend of resistance to antibiotics for ocular infection of *Staphylococcus aureus*, coagulase-negative staphylococci, and *Corynebacterium* compared with 10-years previous: A retrospective observational study

**DOI:** 10.1371/journal.pone.0203705

**Published:** 2018-09-07

**Authors:** Hideto Deguchi, Koji Kitazawa, Kanae Kayukawa, Eri Kondoh, Akiko Fukumoto, Toshihide Yamasaki, Shigeru Kinoshita, Chie Sotozono

**Affiliations:** 1 Baptist Eye Institute, Kyoto, Japan; 2 Department of Ophthalmology, Kyoto Prefectural University of Medicine, Kyoto, Japan; 3 Department of Frontier Medical Science and Technology for Ophthalmology, Kyoto, Prefectural University of Medicine, Kyoto, Japan; Universitatsklinikum Munster, GERMANY

## Abstract

**Objective:**

To retrospectively identify epidemiological trends of infection on the ocular surface and investigate trends of resistance to bacterial antibiotics compared with 10-years previous for *Staphylococcus aureus*, coagulase-negative staphylococci (CNS), and *Corynebacterium* in Japan.

**Materials and methods:**

Bacterial isolate samples were collected from the conjunctival sacs of eyes afflicted with conjunctivitis, keratitis, dacryocystitis, and hordeolum from September 2004 through November 2005 (n = 145 isolates) and September 2014 through November 2015 (n = 195 isolates) at the Baptist Eye Institute, Kyoto, Japan. The prevalence of methicillin-resistant *S*. *aureus* (MRSA), methicillin-resistant CNS (MR-CNS), and fluoroquinolone-resistant *Corynebacterium* were examined, and susceptibility of isolated bacteria to levofloxacin (LVFX), cefmenoxime (CMX), chloramphenicol (CP), erythromycin (EM), vancomycin (VCM), and arbekacin (ABK) were compared between both time periods using the disc susceptibility method.

**Results:**

Over the 10-year period from initial to final examination, the prevalence of MRSA and MR-CNS significantly decreased from 52% to 22% (*P* < 0.05) and from 47% to 25% (*P* < 0.05), respectively, yet there was no change in the prevalence of fluoroquinolone-resistant *Corynebacterium* (60% and 54%; *P* = 0.38). Antibiotic-resistance trend analysis revealed that susceptibility to antibiotics in 2014–2015 was similar to that in 2004–2005. MRSA and MR-CNS were susceptible to CP (88%), VCM (100%), and ABK (100%), while fluoroquinolone-resistant *Corynebacterium* was susceptible to CMX (100%), VCM (100%), and ABK (96%).

**Conclusion:**

The prevalence of MRSA and MR-CNS significantly decreased between the two time periods, yet more than 50% of the *Corynebacterium* isolates were still resistant to LVFX. Although no increase in bacterial resistance to antibiotics was found, a cautionary use of fluoroquinolone eye drops should be considered.

## Introduction

Ocular infections such as conjunctivitis and bacterial keratitis are acute or chronic infections that are often caused by the improper care and cleaning of contact lenses or trauma to the ocular surface, and may result in conjunctival scarring or severe keratitis, ultimately leading to loss of vision [1,2]. Although antibiotic therapy has improved the rates of clinical remission in patients suffering from ocular surface infection [3], microbial resistance to antibiotics continues to be a serious problem that is on the rise and needs to be overcome.

*Staphylococcus aureus* and coagulase-negative staphylococci (CNS), including *Staphylococcus epidermidis*, are important pathogens that reportedly lead to ocular surface infection [1]. MRSA is known to cause severe ocular surface infections that can lead to loss of vision, and our and others’ previous studies have reported that methicillin-resistant *S*. *aureus* (MRSA) and methicillin-resistant coagulase-negative staphylococci (MR-CNS) are highly resistant to various antibiotics [2,4–7]. It has recently been reported that *Corynebacterium* can cause ocular surface infection, even though it is considered to be a non-pathogenic organism [8–12], and fluoroquinolone-resistant *Corynebacterium* has become a growing issue of concern [9,13].

Our and others' studies have reported that vancomycin ophthalmic ointment 1% is effective for the treatment of intractable ocular MRSA and methicillin-resistant *S*. *epidermidis* (MRSE) infections [14,15]. However, it is widely known that the misuse, overuse, or prophylactic use of these new antibiotics can result in antimicrobial resistance, which presents a serious problem [16–18]. In fact, there are now confirmed reports of a resistance to treatment with vancomycin [19]. Thus, we postulate that the antibiotic susceptibility patterns of organisms on the ocular surface, i.e., MRSA, MRSE, and fluoroquinolone-resistant *Corynebacterium*, in specific, might change over numerous years (e.g., 10 years or more). However, there are currently few reports regarding the trend of antimicrobial resistance over a 10-year time period for the treatment of acute bacterial ocular infections [20].

The purpose of this present study was to retrospectively identify epidemiological trends of infection on the ocular surface, and investigate antibiotics compared with 10-years previous.

## Materials and methods

This retrospective study was approved by the Kyoto Ethics Review Board (ERB), Kyoto, Japan (ERB Approval: #1604), and written informed consent was obtained from all subjects in accordance with the tenets set forth in the Declaration of Helsinki.

This single-center study involved 115 eyes of 103 consecutive Japanese outpatients (50 males and 53 females; mean age: 50.4 years) diagnosed with ocular infection between September 2004 and November 2005, and 127 eyes of 126 consecutive outpatients (59 males and 67 females; mean age: 59.6 years) diagnosed with ocular infection at the Baptist Eye Institute, Kyoto, Japan between September 2014 and November 2015. Ocular infections included conjunctivitis, keratitis, hordeolum, dacryocystitis, and other (i.e., blepharitis, cellulitis, and scleritis). Bacterial isolates were identified, accounting for 145 isolates in 2004–2005 and 195 isolates in 2014–2015.

At the onset of ocular infection, bacterial isolates were collected from the conjunctival sac by use of a sterile cotton swab (Seed Swab No. 2; Eiken Chemical Co., Ltd., Tokyo, Japan) with the utmost care not to contaminate ocular samples with organisms on skin. The samples were inoculated in aerobic culture, and then transported to the research laboratory on the day of collection. The bacteria that grew on the aerobic culture plates were identified by gram staining and coagulase testing. Methicillin resistance was determined by anti-oxacillin. The susceptibility for clinical isolates to levofloxacin (LVFX), cefmenoxime (CMX), chloramphenicol (CP), erythromycin (EM), vancomycin (VCM), and arbekacin (ABK) was measured via the disc susceptibility method for MRSA, MR-CNS, and *Corynebacterium*, because these are the antimicrobials that are mainly available in Japan. Briefly, a bacteria isolate was seeded on the agar plate with antibiotics (KB disc^**®**^, Eiken Chemical Co., Ltd., Tokyo, Japan). After overnight culture, the area of inhibited bacterial growth was assessed according to the specific organism as previously reported [21,22]. There were no changes in susceptibility testing compared with 10-years previous. Two eyes with MR-CNS and 5 eyes with *Corynebacterium* in 2014–2015 that did not undergo susceptibility testing were excluded in this analysis.

Strains of bacteria that were repeatedly obtained from the same eye were counted as the same strain. However, in cases with a different antibiotic resistance pattern, the strains of bacteria were counted as a different strain.

### Statistical analysis

Statistical analysis was performed using JMP^®^ Version 12.1 (SAS Institute, Inc., Cary, NC) statistical software. The prevalence of MRSA, MR-CNS, and fluoroquinolone-resistant *Corynebacterium* between the periods 2004 to 2005 and 2014 to 2015 was statistically evaluated using Fisher’s exact test.

## Results

### Background of patients with ocular infection

**Fig 1:**
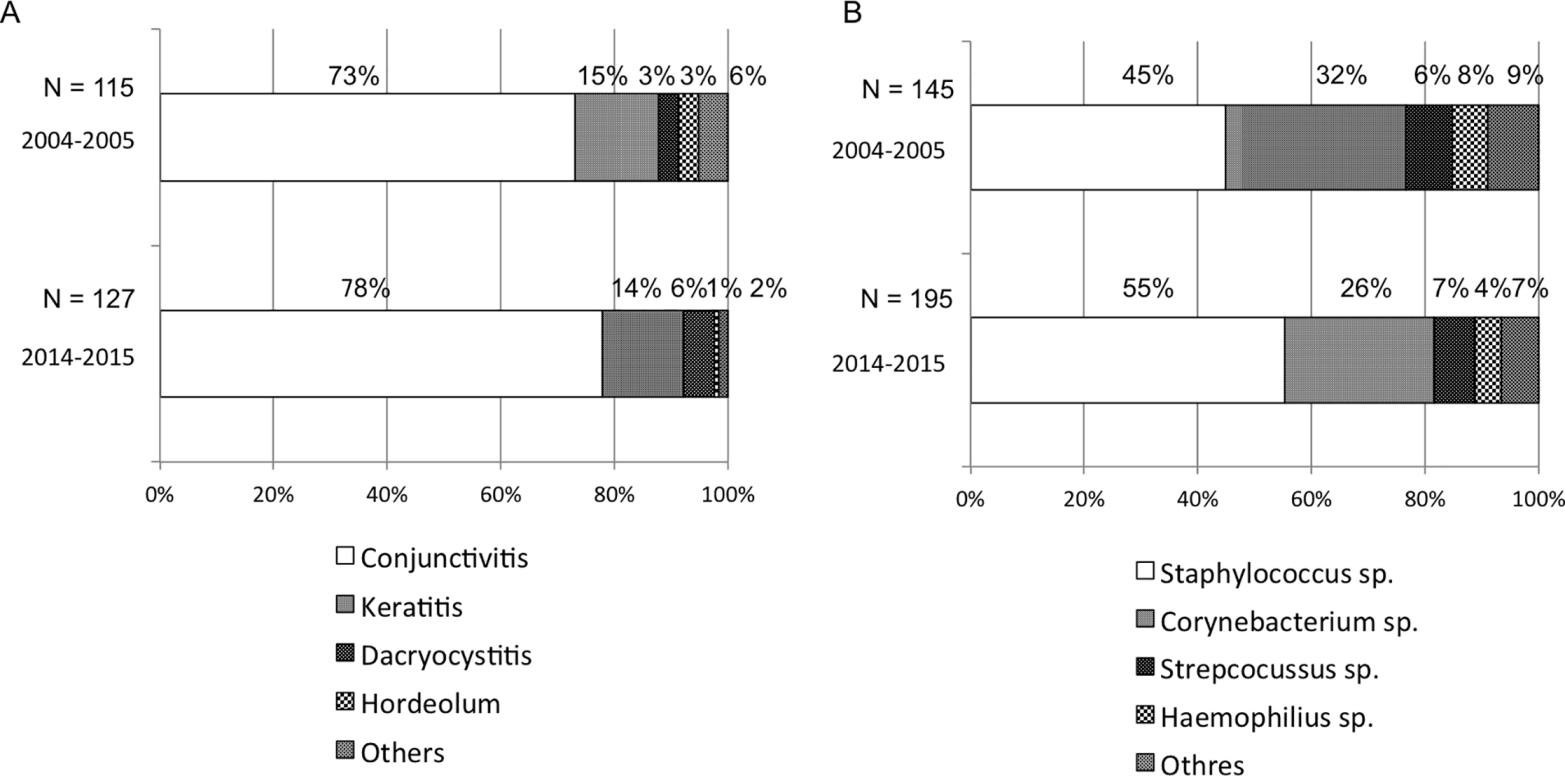
Ocular infections and isolated bacteria obtained from the patients during the periods of 2004–2005 and 2014–2015. (A) Ocular infections observed during both periods. Conjunctivitis was the most commonly observed infection. (B) Isolated bacteria from the patients with ocular infections during both periods. Staphylococcus species were the most common isolates identified in both periods.

Bacterial isolates in patients with acute ocular infections during the period 2004–2005 and 2014–2015 were investigated. Conjunctivitis was the most common manifestation in both periods, accounting for 73% (n = 84) in 2004–2005 and 78% (n = 99) in 2014–2015. Bacterial keratitis was the second most common manifestation, accounting for 15% in 2004–2005 and 14% in 2014–2015. Other ocular diseases encountered included hordeolum and dacryocystitis (Fig 1A). The prevalence of isolated bacteria on the ocular infections revealed that Staphylococcus species were the most common isolates identified in both periods, accounting for 45% (n = 65) in 2004–2005 and 55% (n = 108) in 2014–2015, and *Corynebacterium*, accounting for 32% (n = 46) in 2004–2005 and 26% (n = 51) in 2014–2015. These two isolated bacteria were prevalent in approximately 80% of all the identified bacteria (Fig 1B). These findings suggest that the background diseases and identified bacteria of the patients enrolled in this study were relatively similar between the two observation periods (i.e., 2004–2005 and 2014–2015).

### The trend of bacterial resistance

**Fig 2:**
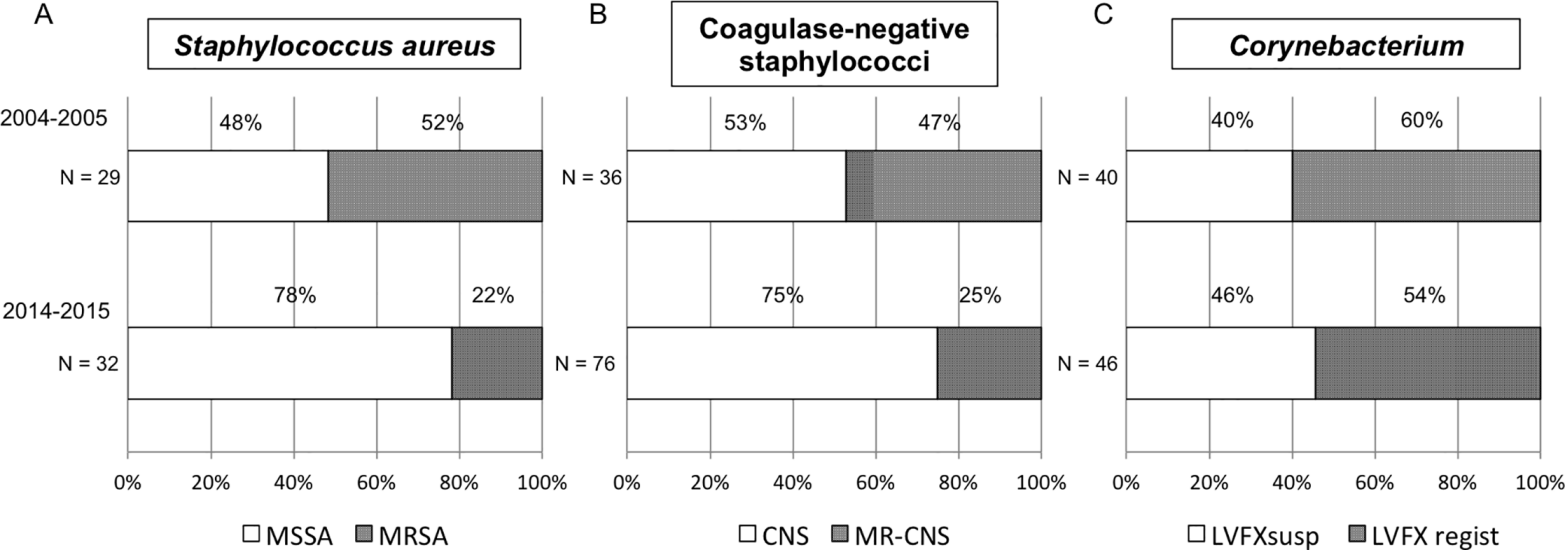
The trend of resistant bacteria during 2004–2005 and 2014–2015. (A) The prevalence of methicillin-resistant *S*. *aureus* (MRSA) in *S*. *aureus* significantly decreased from 52% in 2004–2005 to 22% in 2014–2015 (*P* < 0.05). (B) The prevalence of methicillin-resistant coagulase-negative staphylococci (MR-CNS) in coagulase-negative staphylococci (CNS) significantly decreased from 47% in 2004–2005 to 25% in 2014–2015 (*P* < 0.05). (C) Microbial examination revealed that >50% of *Corynebacterium* in 2004–2005 were resistant to levofloxacin, and that resistance remained through 2014–2015 (*P* = 0.38). MSSA: methicillin-susceptible *S*. *aureus*.

To investigate the trend of bacterial resistance, the pattern of resistance to antibiotics by *Staphylococcus aureus (S*. *aureus)*, coagulase-negative staphylococci (CNS), and *Corynebacterium* in the periods of 2004–2005 and 2014–2015 was examined. During the period of 2004–2005, approximately half of the cases of *S*. *aureus* and CNS were resistant to methicillin [MRSA: 52% (n = 15), MR-CNS: 47% (n = 17), respectively], while 10-years later, the prevalence of MRSA in *S*. *aureus* infections had decreased to 22% (n = 7) and MR-CNS in CNS had decreased to 25% (n = 19), thus illustrating significant reduction rates compared with 10-years previous (*P* < 0.05 and *P* < 0.05, respectively) (Fig 2A and 2B). However, there was no change in the prevalence of fluoroquinolone-resistant *Corynebacterium* during the 10-year time period [i.e., 60% (n = 24) in 2004–2005 and 54% (n = 25) in 2014–2015, respectively; *P* = 0.38] (Fig 2C).

### Susceptibility to antibiotics for MRSA, MR-CNS, and fluoroquinolone-resistant *Corynebacterium*

**Fig 3:**
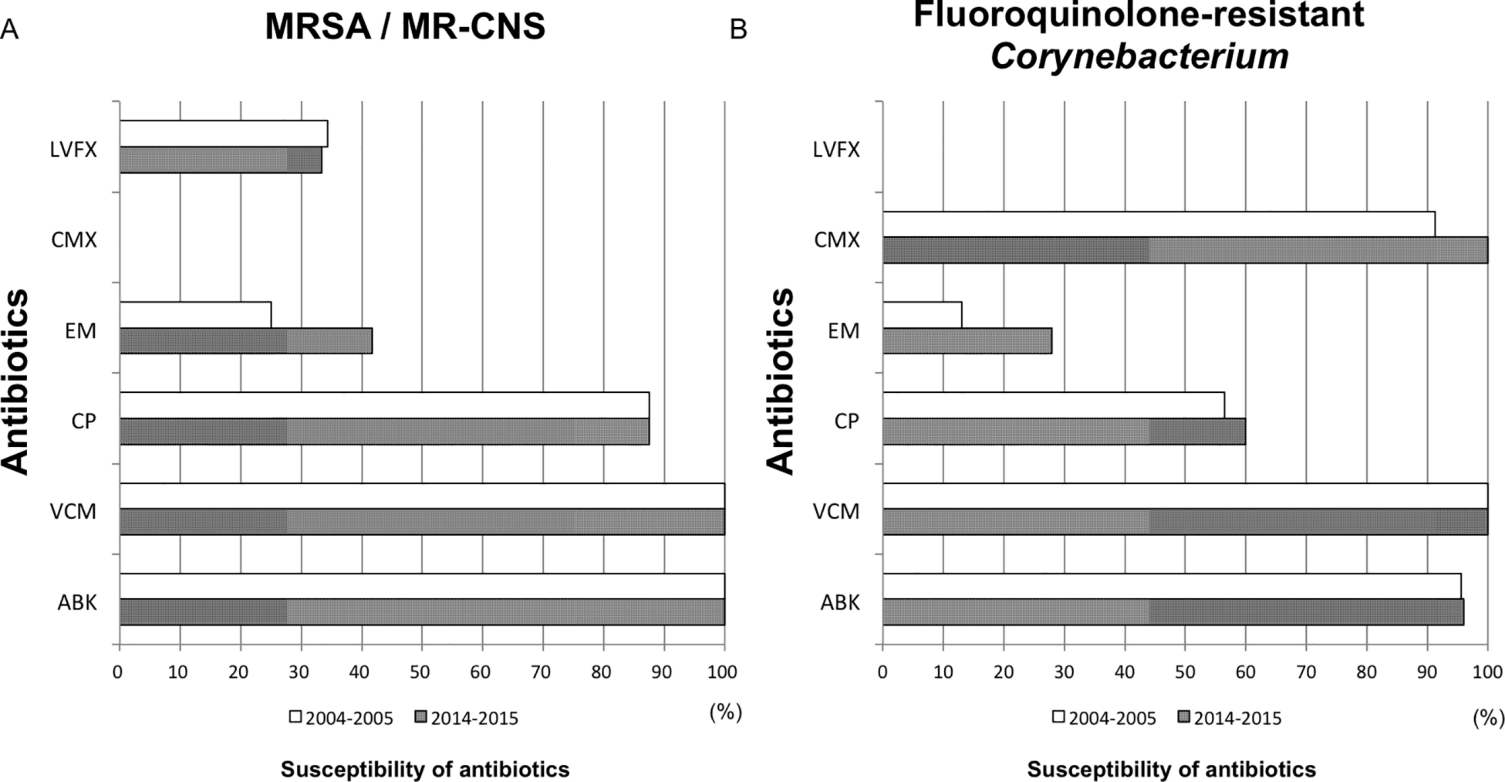
The susceptibility to antibiotics of resistant bacteria during the periods 2004–2005 and 2014–2015. (A) No change of methicillin-resistant *S*. *aureus* (MRSA) or methicillin-resistant coagulase-negative staphylococci (MR-CNS) susceptibility to antibiotics was observed between 2004–2005 (n = 32) and 2014–2015 (n = 26). (B) Fluoroquinolone-resistant *Corynebacterium* susceptibility to antibiotics was similar in both periods (n = 24 in 2004–2005 and n = 25 in 2014–2015).

Trend analysis of antibiotic eye drops for the treatment of MRSA and MR-CNS revealed that the susceptibility to LVFX was 34% in 2004–2005 and 33% in 2014–2015, and that the susceptibility to EM was 25% in 2004–2005 and 42% in 2014–2015. All cases of MRSA and MR-CNS were resistant to treatment with CMX. Susceptibility to CP was 88% in 2004–2005 and 88% in 2014–2015. All MRSA and MR-CNS cases were susceptible to VCM and ABK, thus suggesting that the susceptibility of MRSA and MR-CNS to antibiotics had not changed compared with 10-years previous (Fig 3A). In regard to fluoroquinolone-resistant *Corynebacterium*, the susceptibility to antibiotics in 2014–2015 was nearly similar to that of 2004–2005, yet the susceptibility to EM had increased from 13% to 28%. The susceptibility to CMX was equivalent to that of to ABK and VCM during both periods, however, the susceptibility to CP of fluoroquinolone-resistant *Corynebacterium* was relatively low compared to that of MRSA and MR-CNS (Fig 3B).

## Discussion

In this retrospective study, our findings revealed that the prevalence of MRSA and MR-CNS infections in Japanese subjects with ocular infections has significantly decreased compared with 10-years previous, yet the prevalence of fluoroquinolone-resistant *Corynebacterium* infection has remained the same. In addition, our findings showed no change in the trend of antibacterial resistance in MRSA, MR-CNS, and fluoroquinolone-resistant *Corynebacterium* between the periods of 2004–2005 and 2014–2015.

It is widely known that antimicrobial resistance is rising to high levels globally due to the misuse and overuse of antibiotics, and that the resistance to multiple antibiotics is on the increase. Numerous studies have reported that MRSA contributes to the pathology of various eye diseases, such as refractory corneal ulcer, conjunctivitis, endophthalmitis, orbital cellulitis, and blebitis post glaucoma surgery[2,7,23–31]. We previously reported that subject exposure to medical treatment environments (i.e., hospital facilities and private clinics) accelerates the risk factor of MRSA colonization and MRSA infections [32], and that MRSA keratitis post refractive surgery is one of the severe complications [2,33,34]. A previous study reported that there is an increased tendency in the prevalence of MRSA conjunctivitis in elderly people aged 65 years or older, and that it accounted for 15.3% in 1996, 58.3% in 1997, and 69.8% in 1998 [35]. However, other recent studies have reported that the incidence of MRSA reached its peak in the late 1990s, and has actually been on the decline since the late 2000s [36–38]. The findings in this present study on acute ocular infection revealed that the prevalence of MRSA or MR-CNS infections has significantly decreased compared with 10-years previous, which is similar to the findings in recent reports in other fields of medicine. However, compared to the previous report in 2012 by Hsiao and colleagues [39], which showed that the prevalence of MRSA in ocular *S*. *aureus* infections was 53%, the prevalence of MRSA in our present study was 22%, and thus relatively low. This might be due to the decline of healthcare-associated MRSA (HA-MRSA) that results from the proper use of the antibiotics and strict compliance with standard precautions [40], such as practiced in our institute. However, the distinction between community-acquired MRSA (CA-MRSA) and HA-MRSA was not considered or investigated in this present study.

Increased microbial resistance to fluoroquinolones has been widely reported [13,41,42]. The findings of Chang and associates [41] demonstrated an increase in the resistance rates to fourth-generation fluoroquinolones for both methicillin-susceptible *S*. *aureus* and MRSA. Moreover, it has been recently reported that MRSA has acquired a resistance to VCM, and that VCM-resistant *S*. *aureus* strains are on the increase in certain countries [43,44]. These findings are possible correlated with the increasing popularity of VCM eye drops or ointment since their introduction. Contrary to what we had expected, the findings in this present study demonstrated no change in the resistance level of MRSA or MR-CNS to LVFX, and that they were both susceptible to ABK and VCM, thus suggesting that the trend of MRSA and MR-CNS resistance to various antibiotics during the 10-year time period was stable in the field of ophthalmology, similar to the findings in other fields of medicine. It has been reported that MRSA and MR-CNS maintained a reasonable level of sensitivity to CP, a commonly used empirical topical antibiotic eye drop [4]. Interestingly, CP has higher susceptibility than LVFX and CMX, thus suggesting that it may be selected as a ‘first choice’ treatment for MRSA and MR-CNS, as previously reported [4].

Interestingly, 60% of the *Corynebacterium* isolates in this present study had acquired resistance to LVFX in 2004–2005. It has been reported that *Corynebacterium* is a non-pathogenic organism. However, recent clinical findings have shown the existence of fluoroquinolone-resistant *Corynebacterium* in ocular-surface infections [9,11–13], and in Japan, fluoroquinolone eye drops are commonly used as a ‘first choice’ treatment for ocular infections. Thus, we had feared an increase in the resistance to fluoroquinolones. Investigation of the trend of the prevalence of fluoroquinolone-resistant *Corynebacterium* revealed that half of *Corynebacterium* is resistant to fluoroquinolones, contrary to the decline of the prevalence of MRSA and MR-CNS, even though the prevalence of resistant *Corynebacterium* had not increased compared with 10-years previous. This finding suggests that a cautionary use of fluoroquinolone eye drops should maybe be followed in order to reduce the prevalence of fluoroquinolone-resistant *Corynebacterium*. The susceptibility analysis of the antibiotics showed that fluoroquinolone-resistant *Corynebacterium* had high sensitivity (i.e., more than 90%) to CMX, thus suggesting that CMX might be an effective alternative treatment in cases of conjunctivitis resistance to LVFX.

It should be noted that this study did include some limitations. First, it was a retrospective study, and some of the data without susceptibility testing were excluded. Second, we focused on only MRSA, MR-CNS, and fluoroquinolone-resistant *Corynebacterium* in this study, because approximately 80% of the isolated bacteria were *Staphylococcus*, CNS, and *Corynebacterium*. Thus, further study is necessary to investigate the drug susceptibility for all isolates of ocular infections. Third, CNS and *Corynebacterium* may not have been the cause of some of the infections, as their pathogenic potential is significantly less than that of *S*. *aureus*. However, the ocular samples were collected with utmost care in order to not contaminate those samples with organisms on skin, and we think that this present study showed resistant bacteria of only ocular surface infections.

In conclusion, our findings demonstrated that MRSA, MR-CNS, and fluoroquinolone-resistant *Corynebacterium* had no significant increase of resistance to various antibiotics compared with 10-years previous, while 50% in *Corynebacterium* infections were still resistant to fluoroquinolones. Moreover, we found that in eye-drop treatments, MRSA and MR-CNS organisms were susceptible to CP and that the fluoroquinolone-resistant *Corynebacterium* organism was susceptible to CMX. We hope that our findings in this present study will prove beneficial to empirical antibacterial treatment.
